# Targeted delivery of chemotherapy using HSP90 inhibitor drug conjugates is highly active against pancreatic cancer models

**DOI:** 10.18632/oncotarget.12642

**Published:** 2016-10-13

**Authors:** Egor Bobrov, Natalia Skobeleva, Diana Restifo, Natalya Beglyarova, Kathy Q. Cai, Elizabeth Handorf, Kerry Campbell, David A. Proia, Vladimir Khazak, Erica A. Golemis, Igor Astsaturov

**Affiliations:** ^1^ Program in Molecular Therapeutics, Fox Chase Cancer Center, Philadelphia, PA, USA; ^2^ Histopathology Facility, Fox Chase Cancer Center, Philadelphia, PA, USA; ^3^ Biostatistics and Bioinformatics Facility, Fox Chase Cancer Center, Philadelphia, PA, USA; ^4^ Immune Cell Development and Host Defense Program, Fox Chase Cancer Center, Philadelphia, PA, USA; ^5^ Synta Pharmaceuticals, Lexington, MA, USA; ^6^ Nexus Pharma, Langhorne, PA, USA

**Keywords:** HSP90i drug conjugate, Pancreatic cancer, patient-derived xenografts, SN-38, KPC mouse model

## Abstract

The lack of effective treatment modalities is a major problem in pancreatic cancer (PCa), a devastating malignancy that is nearly universally driven by the “undruggable” KRAS and TP53 cancer genes. Poor tumor tissue penetration is the major source of resistance in pancreatic cancer where chemotherapy is the mainstay of treatment. In this study we exploited the selective tumor-targeting properties of the heat shock 90 protein inhibitors as the vehicle for drug delivery to pancreatic tumor tissues. STA-12-8666 is a novel esterase-cleavable conjugate of an HSP90i and a topoisomerase I inhibitor, SN-38. STA-12-8666 selectively binds activated HSP90 and releases its cytotoxic payload resulting in drug accumulation in pancreatic cancer cells *in vivo*. We investigated the preclinical activity of STA-12-8666 in patient derived xenograft and genetic models of pancreatic cancer.

Treatment with STA-12-8666 of the *KPC* mice (knock-in alleles of *LSL-Kras^G12D^*, *Tp53^fl/fl^* and *Pdx1-Cre* transgene) at the advanced stages of pancreatic tumors doubled their survival (49 days vs. 74 days, *p*=0.008). STA-12-8666 also demonstrated dramatically superior activity in comparison to equimolar doses of irinotecan against 5 patient-derived pancreatic adenocarcinoma xenografts with prolonged remissions in some tumors. Analysis of activity of STA-12-8666 against tumor tissues and matched cell lines demonstrated prolonged accumulation and release of cytotoxic payload in the tumor leading to DNA damage response and cell cycle arrest.

Our results provide a proof-of-principle validation that HSP90i-based drug conjugates can overcome the notorious treatment resistance by utilizing the inherently high affinity of pancreatic cancer cells to HSP90 antagonists.

## INTRODUCTION

Pancreatic adenocarcinoma (PDAC) affects 44,000 individuals yearly in the US [1]. This cancer is almost universally lethal with very limited efficacy of chemotherapy (gemcitabine, nab-paclitaxel, platinum, irinotecan and 5FU) and only nominal 2 weeks survival gain from the addition of anti-EGFR agent, erlotinib, to chemotherapy [2]. Clinical trials addressing this glaring need for expanded portfolio of anti-cancer agents active in PDAC have been unsuccessful [3]. Investigations of anti-angiogenesis [4] or EGF receptor targeting [5] produced responses in some individuals while overall comparisons failed to demonstrate improvements statistically. In part, the sluggish progress in the field is due to the biology of PDAC which is nearly universally driven by “undruggable” and interdependent mutations in KRAS, P53 [6]. In response to the urgent need for transformative treatments in PDAC, screening for potentially effective agents has been streamlined with the use of preclinical models closely representative of clinical PDAC such as primary patient-derived xenografts and genetically engineered mouse models [7, 8]. These clinically predictive models of pancreatic cancer are poised to overcome the deficiencies of the *in vitro* propagated and “plastic-adapted” cell lines [9].

The delivery of cytotoxic chemotherapy to the tumor bed has been the “holy grail” of pancreatic cancer research [10]. Attempts to dismantle the fibroblastic stroma by blocking sonic hedgehog signaling [10] was a disappointing failure in the clinic [11] despite the initial improvement of gemcitabine penetration. Reprogramming to quiescence of the stellate fibroblast cells through application of vitamin D has shown some improvement in drug delivery [12] although the view on the PDAC stroma has shifted to being a restraint of carcinoma growth instead of being a physical chemotherapy “barrier”[13–15]. We chose to pursue a different route by exploiting a natural dependency of cancer cells [16, 17], and PDAC in particular [18–20], on the activity of HSP90. Here, we report the results of preclinical evaluation of STA-12-8666, a small molecule drug conjugate in which a selective HSP90 inhibitor is paired with a topoisomerase I inhibitor SN-38 via an esterase-cleavable chemical linker [21, 22]. Our results provide evidence for highly promising STA-12-8666 activity against pancreatic cancer models.

## RESULTS

### STA-12-8666 is a dual HSP90 and topoisomerase I inhibitor

**Figure 1:**
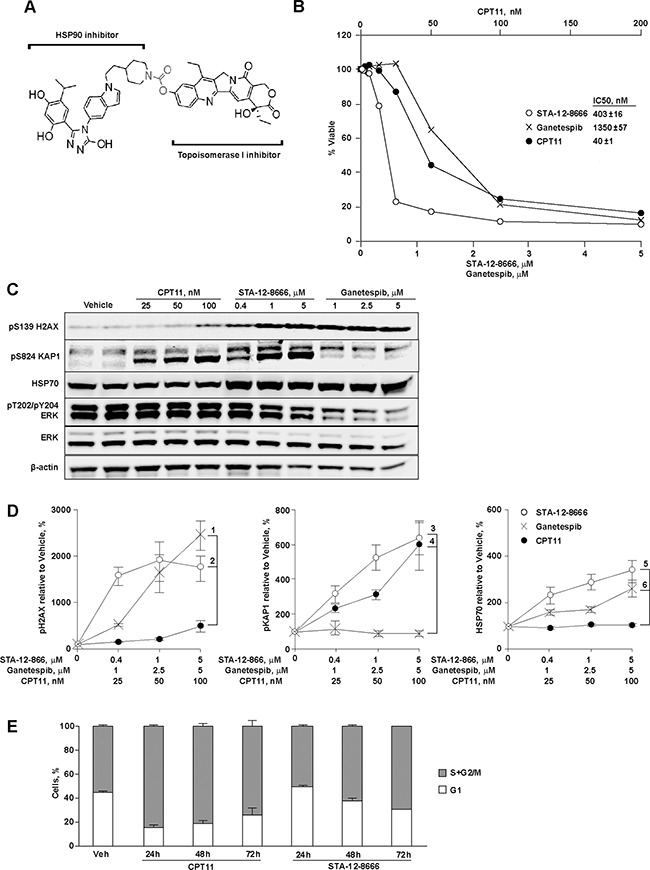
STA-12-8666 is a cleavable small molecule conjugate with HSP90 and topoisomerase I inhibitory activities **A**. STA-12-8666 chemical structure incorporating an HSP90 targeting moiety, a cleavable linker (in red) and SN-38, a topoisomerase I inhibitor. **B**. Sensitivity of KPC murine pancreatic adenocarcinoma cells to CPT11, STA-12-8666 and ganetespib. Shown is percent of viable cells relative to vehicle at 72 hours. *Insert*, IC50 values. **C**. Representative Western blot of KPC cell lysates following treatment with CPT11, STA-12-8666 and ganetespib at the indicated concentrations for 24 hours. **D**. Quantification of Western blot data. Averaged bands intensities for pH2AX, pKAP and HSP70 from 3 independent repeats were normalized to actin; data represent mean±SEM. Spearman's correlation *p* values for comparisons are as shown: 1) 10^−4^; 2) 0.004; 3) <10^−3^; 4) 0.004; 5) 0.01; 6) *NS*, non-significant. **E**. Inhibition of topoisomerase I with CPT11 (p=0.04 vs. vehicle) and STA-12-8666 (*p*=0.0002 vs. vehicle) causes arrest of KPC cells in G2/M phase. Nuclear DNA contents were assessed following treatment at the concentration of 100 nM for indicated periods of time. In all graphs, data are represented as mean ±SEM of three independent repeats. Statistical significance estimated by linear mixed model of the effect over time; there was no difference between CPT11 and STA-12-8666 (*p*=0.25).

Heat shock protein 90 is a critical chaperone to maintain the integrity of the oncogenic signaling in cancer [16, 17]. The activated HSP90 protein in complexes with other co-chaperons has higher affinity for HSP90 selective inhibitors [17]. By design, STA-12-8666 is a dual inhibitor of topoisomerase I (TOP1) and HSP90 (Figure 1A). However, the activity of SN-38 against TOP1 can only be exerted when SN-38 is released from the chemical bond by the cellular esterase activity [21, 22]. Using a mouse pancreatic carcinoma cells derived from the KPC (genotype *LSL-Kras^G12D^;Tp53^f/f^;Pdx1-Cre*) tumor at early passages (P8-10), we tested STA-12-8666 activity against both cellular targets. Assessment of an *in vitro* cytotoxicity of STA-12-8666 and structurally comparable inhibitors of HSP90 (ganetespib) or TOP1 (camptothecin, CPT11) demonstrated that the conjugate has approximately 10-fold higher cytotoxic concentration of 50% (IC50) in comparison to CPT11 (Figure 1B). We believe this difference is related to STA-12-8666 being a pro-drug, so that the cytotoxicity of the conjugate is exerted over time upon release of SN-38 following cleavage of the ester bond (Figure 1A). Using established IC50 values, we then compared the effects of STA-12-8666, ganetespib and CPT11 on their intended targets in KPC cells following 24 hour of drug exposure. STA-12-8666 robustly induced expression of pS139-H2AX associated with DNA damage response (Figure 1C). Furthermore, expression of the S824-phosphorylated form of KAP1 (Figure 1C, 1D), which is an established target of ATM in response to DNA strand breaks [23, 24] and a biomarker of TOP1 inhibition by STA-12-8666 [25] has been significantly upregulated in CPT11 and STA-12-8666-treated cells. This was not observed in the vehicle or HSP90 inhibitor treated cells suggesting a direct activity of STA-12-8666 to induce DNA damage response. Conversely, increased expression of HSP70, a biomarker of HSP90 inhibition [16], was induced by STA-12-8666 at the levels comparable to a selective HSP90 inhibitor, ganetespib, used here as a positive control. The release of SN-38 from the conjugated STA-12-8666 compound takes a slow course due to the requirement for cellular esterase activity for the cleavage of the carbamoyl linker: the observed accumulation of KPC cells arrested in G2/M-phase of the cell cycle *in vitro* was notable only after 48 hours of incubation with STA-12-8666 as opposed to a more rapid effect of CPT11 (Figure 1E).

### STA-12-8666 improves survival in mice with spontaneous pancreatic carcinoma

**Figure 2:**
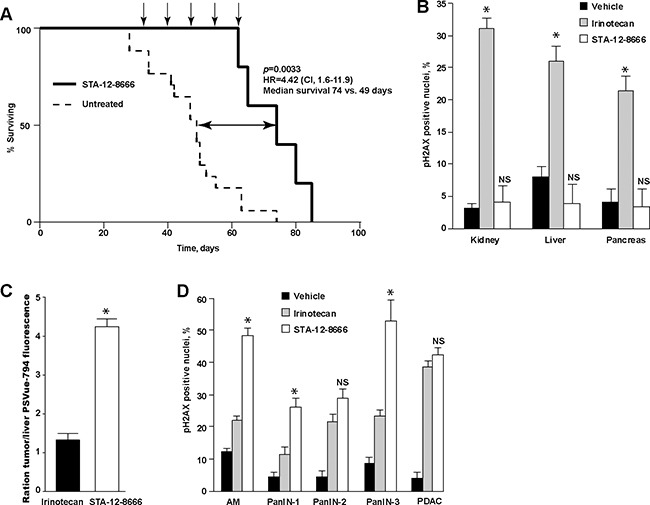
Treatment with STA-12-8666 extends the survival of KPC mice from pancreatic cancer **A**. KPC mice treated with STA-12-8666 (n = 6) survive longer in comparison to control KPC mice (n = 9). The survival was estimated by the Kaplan-Meier method. *Arrows*, drug administration. **B**. Treatmentwith STA-12-8666 does not produce DNA damage in benign tissues in comparison to the equimolar dose of irinotecan. Data represent mean±SEM of percent of nuclear pH2AX-positive cells in kidney, liver and pancreas tissues on day 7, n=3 mice in each group. **C**. Treatment with STA-12-8666 increases phosphatidylserine expression in pancreatic tumors. Shown are mean±SEM fluorescence of PSVue-794 in pancreatic tumors normalized to the liver of same animal, n=2 per group. **D**. Percent of pH2AX-positive nuclei in acinar-to-ductal metaplasia (ADM), pancreatic intraepithelial neoplasm (PanIN) grades 1, 2, 3 and pancreatic adenocarcinoma (PDAC) in KPC mice 7 days following a single dose of vehicle, irinotecan (50 mg/kg) or STA-12-8666 (150 mg/kg). Statistical significance in (B) and (D) was estimated using a mixed effects linear regression model (controlling for within-mouse correlation): *, *p*<0.05 for comparisons of CPT11 and STA-12-8666; *NS*, non-significant.

*KPC* mice carrying a knock-in allele of *LSL-Kras^G12D^* and a “floxed” allele of *Tp53^fl/fl^* in combination with the pancreas-selective *Pdx1-Cre* transgene is a commonly used genetic murine model for human pancreatic cancer [26] which recapitulates the cardinal features of the human disease including resistance to chemotherapy and development of dense desmoplastic stroma surrounding the carcinoma cells. The activation of oncogenic *Kras^G12D^* expression and deletion of *Tp53* in these animals is occurring during fetal development due to the constitutive activity of *Pdx1-Cre* transgene [27]. Untreated animals rapidly succumb to the locally advanced and metastatic pancreatic carcinoma with median survival in our colony around 7 weeks of age. Treatment with STA-12-8666 starting at weeks 5 of age doubled the survival of KPC animals (Figure 2A, 49 days in vehicle vs. 74 days in STA-12-8666 group, Kaplan-Meyer log rank test, *p*=0.008). The animals experienced no apparent toxicity during the treatment. Histochemical evaluation of the liver, kidney or pancreatic tissues collected from 7 week old tumor bearing mice 7 days following administration of a single dose of STA-12-8666 or an equivalent dose of irinotecan demonstrated significantly lower incidence of pS139-H2AX-positive nuclei (indicative of DNA-damage response [28]) in the STA-12-8666-treated animals in comparison to the irinotecan group in keeping with the established tissue toxicity of SN-38 (Figure 2B, Supplementary Figure S1). We assessed apoptosis using an intravenously administered infrared fluorescent phosphatidylserine label, PSVue-794 [29], and compared tumor-to-liver signal intensity *ex vivo*. In keeping with our enumerations of DNA damage response (Figure 2B), there was nearly 4-fold better apoptosis induction selectivity of STA-12-8666 towards the pancreatic tumor in comparison to irinotecan (Figure 2C, Supplementary Figure S2).

Histological evaluation of pancreatic tumors demonstrated increased expression of nuclear pS139-H2AX in the pre-neoplastic lesions (acinar-to-ductal metaplasia, ADM, pancreatic intraepithelial neoplasm, PanIN) and in the invasive adenocarcinoma (PDAC) following a single dose of STA-12-8666 or irinotecan (Figure 2D, Supplementary Figure S1) compared to vehicle.

### Activity of STA-12-8666 against human pancreatic adenocarcinoma xenografts

**Figure 3:**
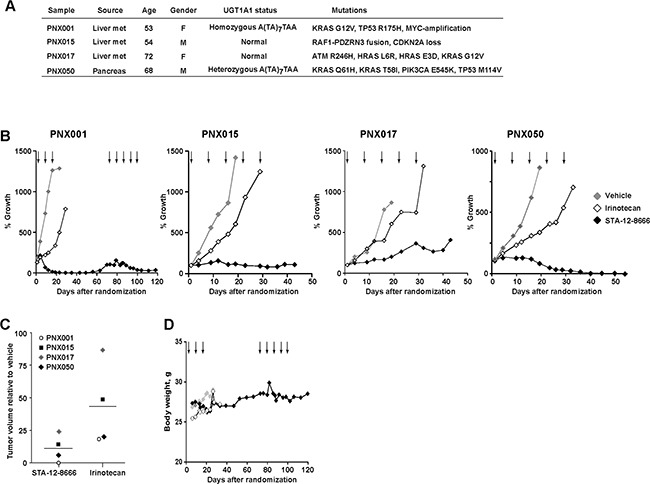
Efficacy of STA-12-8666 against patient-derived pancreatic adenocarcinoma xenografts **A**. Characteristics of patient-derived xenografts used in the study. **B**. Tumor growth curves of PNX001, PNX015, PNX017 and PNX050 xenografts. **C**. PDX tumor volume at 3 weeks on treatment with STA-12-8666 (150 mg/kg), irinotecan (50 mg/kg) relative to vehicle. **D**. Averagedbody weight of mice bearing PNX001 PDX per each treatment group. *Arrows*, intravenous drug administrations. Data represent averaged tumor volumes of at least 3 xenografts from each treatment group.

We further validated the activity of STA-12-8666 on a representative panel (Figure 3A) of early passage (P2-4) patient-derived pancreatic adenocarcinoma xenografts. These tumors were propagated exclusively *in vivo* and were directly obtained from pancreatic cancer surgical samples. All but one tumor carried KRAS and TP53 mutations. One model, PNX001, was characterized by homozygosity for the hypomorphic allele of UDP-glucuronosyltransferase 1 (UGT1A1) important for detoxification of SN-38 [30], and PNX050 was heterozygous for this allele. Without exception, equimolar doses of STA-12-8666 (150 mg/kg i.v. once a week) showed markedly superior activity in comparison to irinotecan (50 mg/kg) given on the same schedule (Figure 3B). In PNX001 the effect was dramatic, with complete tumor regression after 3 weekly injections in all treated animals. Complete responses were also observed in the PXN050 model and lasted more than one month (Figure 3B). Comparing tumor sizes 3 weeks post irinotecan or STA-12-8666 treatment clearly illustrate the superior efficacy obtained with the drug conjugate (Figure 3C). As in our experiments with the transgenic pancreatic cancer model (Figure 2), we did not observe any apparent toxicity in animals treated with STA-12-8666 as reflected in their stable body weight (Figure 3D) and behavior.

**Figure 4:**
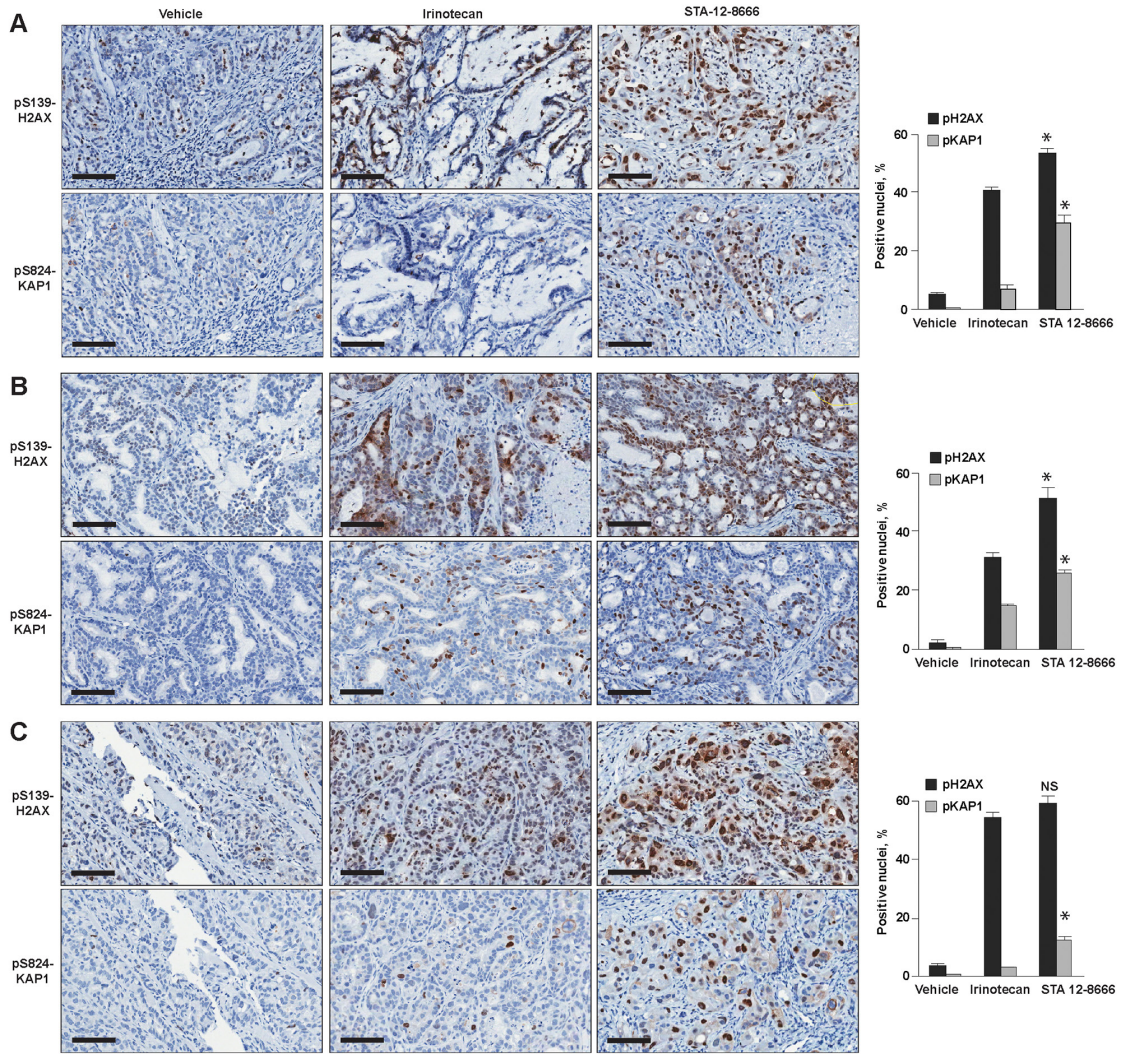
Immunohistochemical analysis of patient-derived xenografts treated for 72 hours with vehicle, Irinotecan and STA-12-8666: **A**. PNX001, **B**. PNX015 and **C**. PNX017. Quantified results for nuclear pH2AX and pKAP1 are shown next to representative immunohistochemistry images. Data represent mean ±SEM of at least 3 xenografts from each treatment group. Statistical significance in biomarker expression between CPT11 and STA-12-8666 treated samples was estimated using ANOVA for pairwise comparisons: *, *p*<0.05 for comparisons of CPT11 and STA-12-8666; *NS*, non-significant.

Histological analyses of pancreatic carcinoma xenografts treated with a single injection of irinotecan or STA-12-8666 demonstrated robust induction of DNA damage response biomarkers consistent with its superior *in vivo* efficacy (Figure 4).

## DISCUSSION

Treatment of pancreatic cancer is challenging due to the lack of effective agents and the resistance to chemotherapy attributable to a greatly reduced blood supply and the desmoplastic stromal response which prevents achieving therapeutically active concentrations for the most administered agents in the tumor bed. Strategies to enhance drug penetration and retention in the tumor bed have been actively pursued in the clinic and proved to be effective as in the case of nab-paclitaxel, the first FDA-approved drug conjugate made of paclitaxel bound to albumin particles [31]. A number of other mechanisms to improve the delivery of cytotoxic payload are under investigation including antibody-SN-38 conjugates, sacituzumab [32], milatuzumab [33] and epratuzumab [34], and polymer-conjugated irinotecan [35]. Despite some advantages of controlled release of the cytotoxic payload, such delivery strategies are limited by the paucity of vascular supply in the pancreatic tumor and the large molecular weight of the protein carriers which restricts their ability to penetrate to the tumor. The strategy that has been pursued here is novel in its design of a small hybrid molecule (MW=880 Da) that is capable of penetrating the tumor and, importantly, also retained in the malignant cells through interaction with activated Hsp90 [22].

To validate the preclinical efficacy of STA-12-8666, we employed a platform of KPC mice genetically induced to generate rapidly progressive pancreatic carcinoma and a series of patient pancreatic tumor tissues explants. Our results provide the proof-of-principle indication that small molecule targeting of proteins selectively activated in cancer but not in normal tissues may be a highly effective drug delivery strategy. HSP90 is an ideal candidate for such an approach [17]. Besides SN-38 used in STA-12-8666 conjugate as a “partner” drug, other cytotoxic or imaging compounds can be covalently attached to the HSP90-inhibitory moiety to provide a versatile delivery system tailored to essentially any cancer type.

The observed activity of STA-12-8666 against pancreatic carcinoma models is impressive. Not only did we observed extended survival of the KPC tumor-bearing mice, but also complete and sustained regression of xenografted PDX tumors suggesting the ability of the conjugate to be selectively retained in pancreatic cancer and to be gradually released to produce an irreversible DNA damage via TOP1 inhibition. In contrast to conventional irinotecan, STA-12-8666 had minimal effect on the benign tissues in mice as confirmed by the absence of DNA damage response or apoptosis induction with in vivo imaging (Figure 2). We also note that in our recent study, we tested these PDX models against conventional chemotherapy currently in use in pancreatic cancer [8]. The tumor growth inhibition demonstrated here with STA-12-8666 by far supersedes the effect of most active chemotherapy showing only about 50% tumor growth inhibition (mitomycin C, gemcitabine, nab-paclitaxel, oxaliplatin and irinotecan among tested drugs). Of interest, in two PDX models showing the greatest sensitivity to STA-12-8666 and irinotecan (PNX001 and PNX050 in Figure 3), we identified polymorphisms of UGT1A1 gene which is responsible for glucuronide-dependent inactivation of SN-38. *In vivo*, the hepatic conversion of CPT11/irinotecan to SN-38 is responsible for the dose-limiting toxicity of the drug [36]. Similarly to CPT11, STA-12-8666 is being activated by cellular esterase thus releasing the SN-38 payload. However, the key difference between the two pro-drugs is that STA-12-8666 accumulates preferentially in the tumor cells because of the bond with the HSP90-targeting moiety. This differential uptake by the tumor may be useful for the future clinical testing of STA-12-8666 as the same polymorphisms are retained in the patients’ tumors which may increase their susceptibility to topoisomerase inhibition with SN-38. Given the absence of any significant organ toxicity of STA-12-8666 at the doses used in rodents, pancreatic and other cancers arising in the carriers of Gilbert syndrome allele of UGT1A1 [37] could be highly sensitive to STA-12-8666.

In summary, we provide preclinical evidence that the efficacy of the existing chemotherapy agents against pancreatic cancer can be dramatically improved by preferential delivery via our novel HSP90 inhibitor drug conjugate platform. Cardinal features of this innovative system include small molecules size, high bioavailability and prolonged retention in the tumor, as well as lack of organ toxicity resulting in a remarkable therapeutic index which will need to be validated in the future clinical trials of STA-12-8666.

## MATERIALS AND METHODS

### Animals, xenografts and cell lines

For the spontaneous pancreatic cancer model, crosses between *Pdx1-Cre* transgenic mice (JAX mice, 014647) and mice carrying a single *LSL-Kras^G12D^* knock-in allele (JAX mice, 019104) were used. Both of these mouse lines were maintained on C57BL/6 genetic background and subsequently crossed to the C57BL/6 congenic *Trp53^fl/fl^* conditional knockout mice (JAX mice, 008462) to generate *Pdx1-Cre;Trp53^fl/fl^* and *LSL-Kras^G12D^*;*Trp53^fl/fl^* breeding pairs, respectively. Their progenies develop a spectrum of pancreatic tumors as the result of activated oncogenic *Kras^G12D^* allele in combination with Cre-induced ablation of *Tp53* (KPC mice [7]). For xenograft experiments, immunodeficient *C-B17.scid* mice were purchased from the in-house colony. All animals were maintained in our established breeding colony at FCCC. Mice were maintained on Purina breeder chow (LabDiet, St. Louis MN) and received water *ad libitum*. All animal experiments were conducted in compliance with the FCCC Animal Care and Use Committee guidelines and with the NIH Guide for the Care and Use of Laboratory Animals.

The murine pancreatic adenocarcinoma cell line was obtained from a KPC mouse tumor and propagated for 6 passages by the Fox Chase Cancer Center Cell Culture Facility. Frozen stocks of KPC cells were expanded in RPMI-1640, supplemented with 10% fetal bovine serum, 2 mM L-glutamine, 25 ug/ml Insulin and 100 ug/ml penicillin/streptomycin. Patient derived xenografts (PDX) were derived via subcutaneous implantation of surgically obtained pancreatic adenocarcinoma tissue fragments. Frozen stocks of shredded, passage 3 PDX tissues were used for the efficacy studies.

### Antibodies and reagents

The following antibodies were used for Western blots: phospho-H2AX (pSer139, MABE205, Millipore), phospho-KAP1 (pS824, 70369, Abcam), Akt (2920, Abcam), HSP70 (ADI-SPA-820-D, Enzo Life Sciences); antibodies for phospho-Akt (pSer473, #3787), ERK1/ERK2 (#9107), phospho-ERK1/ERK2 (pThr202/pTyr204, #9101), β-actin (#4967), α-tubulin (#2125), EGFR (#2646), phospho-EGFR (pTyr1173, #4407) and PARP (#9542) were purchased from Cell Signaling. STA-12-8666 was supplied by Syntha Pharmaceuticals.

### Cell viability assays

KPC cells were plated in 96-well plates in triplicate at a seeding density of 900 cells/well. After 24 hr, the cells were treated with various concentrations of the drugs. DMSO solvent without drug served as a negative control. After 72 hr of incubation, cells were analyzed for viability using CellTiter Blue Assay (Life Technologies) to determine the inhibitory concentration of 50% (IC50) values.

### Cell cycle assay

KPC cells were seeded on 2×6-well plates at density of 30,000 cells per well in RPMI-1640 supplemented with 1% FBS, 2 mM L-glutamine, 25 ug/ml Insulin and 100 ug/ml penicillin/streptomycin. After 24 hr, the cells were treated with CPT11 and STA 12-8666 at equimolar concentrations (100 nM). Cell-cycle analysis was performed 24, 48 and 72 hr after CPT11 and STA 12-8666 treatment by flow cytometry analysis of DNA content (Guava Cell Cycle Assay). Cells fixed with 70% ethanol overnight at -20°C were washed in PBS and resuspended in Guava Cell Cycle Reagent for Flow Cytometry (4700-0160, Millipore). After 30 min at room temperature in the dark, analysis was performed on a Guava EasyCyte flow cytometer (Millipore) and data were analyzed using Guava CytoSoft 5.3 software. Averaged cell-cycle profiles from three biological repeats were plotted as histograms.

### Western blot

Mouse and human PDX tumor tissues were lysed with T-PER reagent (78510, Thermo Scientific) containing double concentration of Halt™ protease and phosphatase inhibitors (78445, Thermo Scientific). For Western blot, KPC cells were seeded on 6-well plates at density of 30,000 cells per well in RPMI-1640 supplemented with 1% FBS, 2 mM L-glutamine, 25 mg/L Insulin and 100 mg/L penicillin/streptomycin. Next day, the cells were treated with indicated drugs for 24 hours and lysed with RIPA lysis buffer (Santa Cruz Biotechnology) containing Halt™ protease and phosphatase inhibitors (Thermo Scientific). Protein concentrations were measured using BSA assay. Western blot membranes were scanned using Odyssey infrared imaging reagents including blocking buffer and secondary antibodies (LI-COR).

### Treatment of KPC mice

In the KPC survival experiment, mice were prospectively enrolled into drug treatment cohorts at the age of 5 weeks. Saline vehicle (n=9) or STA-12-8666 (n = 6, 150 mg/kg) were given as retro-orbital vein injections weekly for 5 weeks. Mice were sacrificed if moribund, in distress or >20% weight loss. Survival curves were plotted and median survival time for both cohorts was calculated. In dedicated tissue studies, 6 week old KPC mice were treated with single doses of STA-12-8666 (150 mg/kg), irinotecan (50 mg/kg) or saline vehicle via retro-orbital vein injection. On day 7, tumor and organ tissues were evenly divided in 3 parts to obtain lysates, fixed in formalin or frozen.

### Histology

Formalin fixed and paraffin embedded tissues were cut in serial 5 micron sections and H&E stained. Pancreata with identifiable pancreatic adenocarcinoma and pancreatic intraepithelial neoplasm (PanIN) lesions and organ tissues were further stained using immunoperoxidase ABC kit (Vector Laboratories) and indicated antibodies. Stained slides were scanned using ScanScope CS scanner (Aperio) and selected images were analyzed using the ImageScope software. For pKAP1 and pH2AX, quantification of the percentage of stained nuclei was done using nuclear mask algorithm. Results of the quantification were plotted as histograms.

### Xenograft experiments

Cryopreserved in DMSO PDX tumor fragments were quickly thawed, washed in RPMI and resuspended in 1:1 mix of RPMI and Matrigel (Corning) on ice until the moment of implantation. Tumor fragments were subcutaneously injected using 1 ml syringe and 18G 1½ needle in both flanks of 5-8 week old *C-B17.scid* mouse. Animals with established tumors (around 150 mm^3^) were randomly divided to receive STA 12-8666 at 150 mg/kg (n = 5), irinotecan at 50 mg/kg (n = 5), or vehicle (n = 5) via retro-orbital vein injections once a week. Tumors were measured at least two times a week. Tumors and animal weight were measured with a digital caliper two times per week. Tumor volumes were calculated using the modified ellipsoid formula as described [38] until the maximum size of 1500 mm^3^ or if animals exhibited distress, >20% weight loss or if tumors ulcerated in which case mice were humanely euthanized. In dedicated experiments, assessment of PDX tissues was done on day 7 after the drug administration.

### Phosphatidylserine imaging using PSVue molecular probe

For in vitro studies, KPC cells were plated in 96-well plates at a seeding density of 900 cells/well. Next day, the cells were treated with various concentrations of the drugs as indicated for 24 hours. PSVue-794 or control fluorophore were applied as per the manufacturer's protocol (Molecular Targeting Technologies) and analyzed on Odyssey infrared imaging system (LI-COR). For PSVue-794 in vivo imaging experiments, 6 week old KPC mice were treated with STA-12-8666 at 150 mg/kg or irinotecan at 50 mg/kg. On day 7, mice were injected via retro-orbital vein with 150 μl of 1mM PSVue-794, fluorescence and white light images were acquired using IVIS Spectrum imager (PerkinElmer, Hopkinton MA), with excitation = 750nm, emission = 800nm, binning = 1, FOV setting = C, lamp level = high, and exposure times of 5-15 sec, depending on the signal strength. Infrared signal in the tumor was quantified relative to the fluorescence signal in the liver from the same animal. The control animals received the same dose of untargeted PSVue control probe which showed only faint background signal.

## SUPPLEMENTARY FIGURES


