# Supplementary material for: Targeted delivery of chemotherapy using HSP90 inhibitor drug conjugates is highly active against pancreatic cancer models
Source: Oncotarget. 2016 Oct 13;8(3):4399–409. doi: 10.18632/oncotarget.12642 (PMC5354841; doi:10.18632/oncotarget.12642)
Supplement: Supplementary file 1 [file oncotarget-08-4399-s001.pdf]

Targeted delivery of chemotherapy using HSP90 inhibitor drug conjugates is highly active against pancreatic cancer models

SUPPLEMENTARY FIGURES

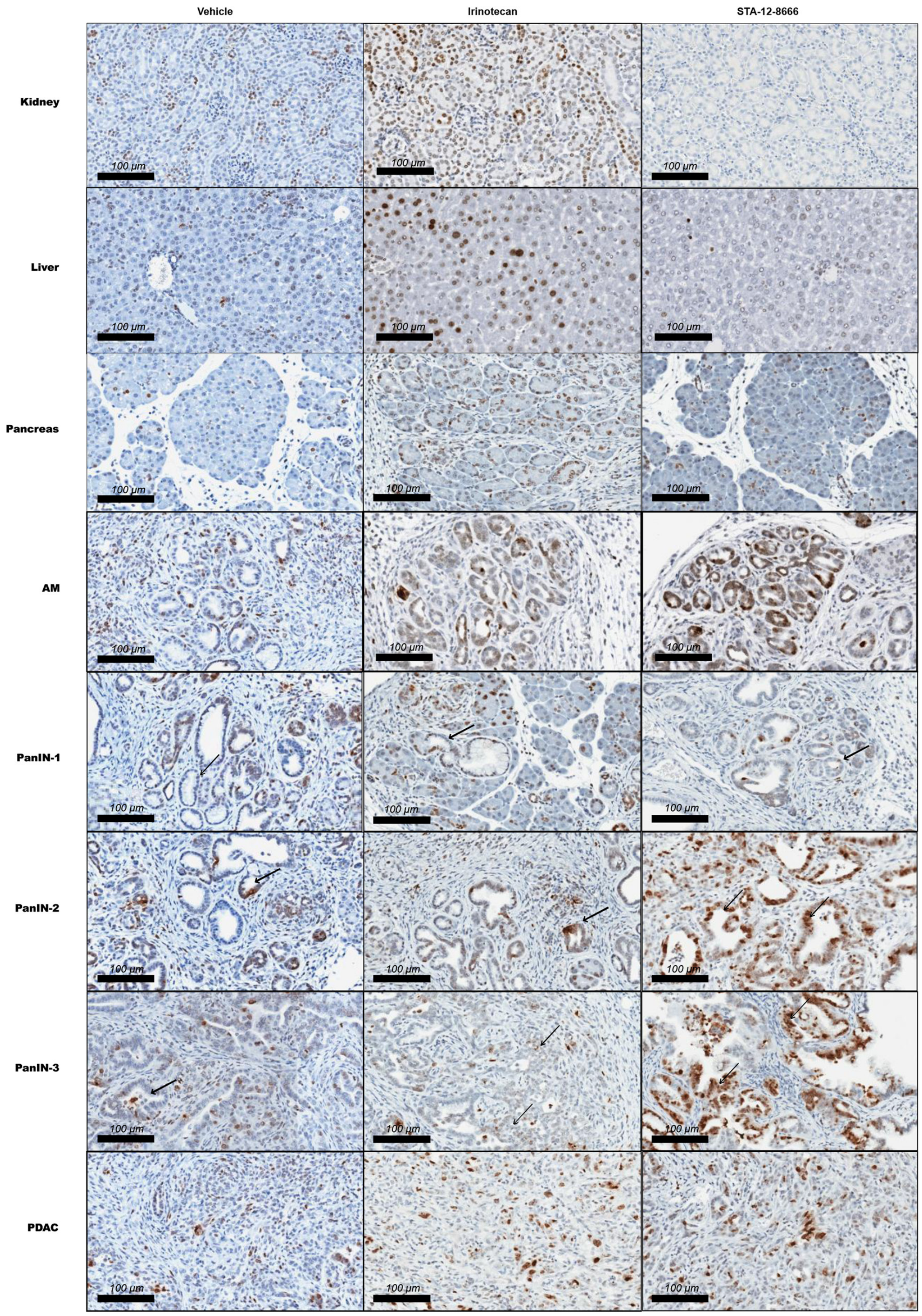

Supplementary Figure S1: Representative immunostaining for pH2AX in kidney, liver, pancreas and different stages of pancreatic cancer progression in KPC mice treated with vehicle, Irinotecan and STA-12-8666. *Abbreviations*: AM, acinar metaplasia; PDAC, pancreatic adenocarcinoma.

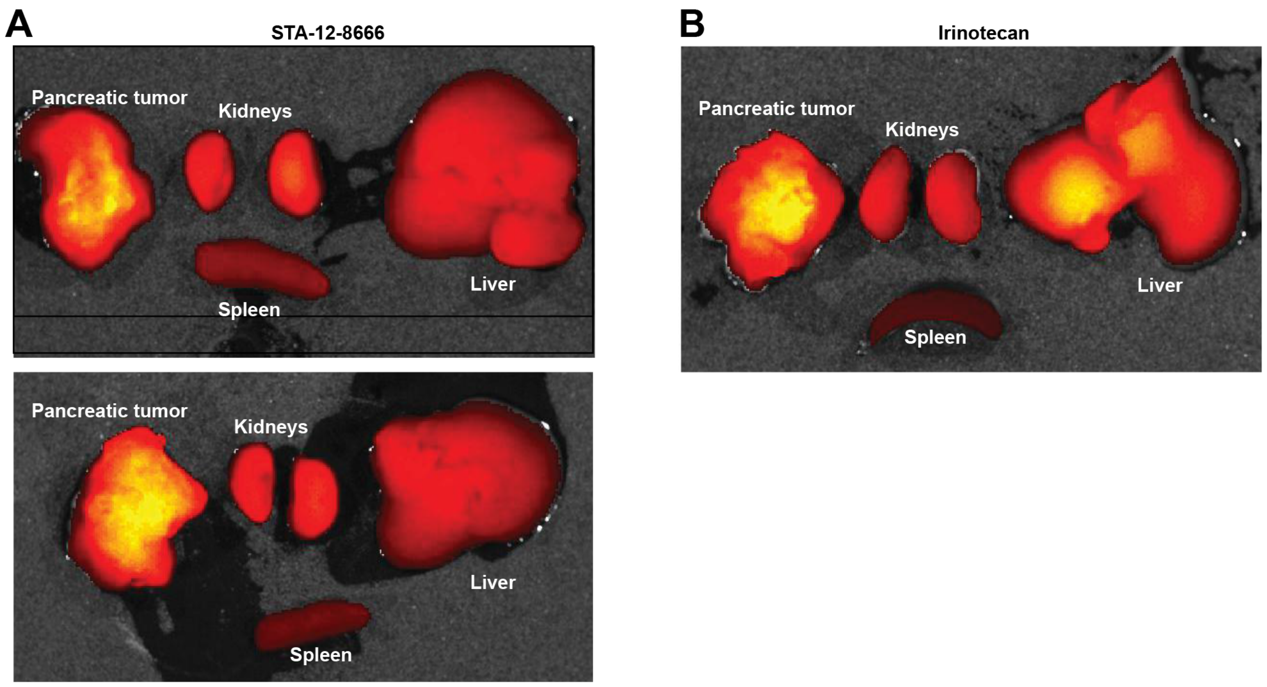

Supplementary Figure S2: Ex vivo infrared images of phosphatidylserine using molecular probe PSVue-794 (yellow glow) in animals treated for 7 days with a single dose of STA-12-8666 at 150 mg/kg **A.** or irinotecan at 50 mg/kg **B.** Note higher level of apoptosis biomarker phosphatidylserine in the liver of irinotecan-treated animal compared to STA-12-8666.
